# Validation of the revised Compound PsyCap Scale (CPC-12R) and its measurement invariance across the US and Germany

**DOI:** 10.3389/fpsyg.2022.1075031

**Published:** 2022-12-22

**Authors:** Timo Lorenz, Leonie Hagitte, Priscilla Rose Prasath

**Affiliations:** ^1^Department of Psychology, MSB Medical School Berlin, Berlin, Germany; ^2^Department of Counseling, University of Texas at San Antonio, San Antonio, TX, United States

**Keywords:** validation, measurement, multigroup confirmatory factor analyses, positive psychology, cross-cultural validity, psychological capital, confirmatory factor analyses (CFA)

## Abstract

The purpose of this study was to validate the English and German versions of the revised Compound PsyCap Scale (CPC-12R) in a US-sample (*n* = 385) and a sample from Germany (*n* = 202). The 12-item CPC-12R exhibited the anticipated factorial structure with an excellent model fit in both samples and associations to other constructs concurred with previous findings. A specific aim was to examine the measurement invariance of the CPC-12R across the two countries. Scalar measurement invariance was established. Overall, these findings suggest that the CPC-12R is an economic, valid, reliable, and applicable tool in the US and Germany to assess psychological capital (PsyCap). The scalar measurement invariance highlights the importance of taking cultural background and possible pitfalls for cross-cultural research into account for future PsyCap research.

## 1 Introduction

Psychological capital (PsyCap) is a construct in positive psychology, which includes different positive psychological resource capacities. Luthans et al. (2006) state that the resource capacities must meet certain criteria to be included in PsyCap. Among other criteria, the capacities must be *state-like* and therefore open for development. From these states, four constructs are proposed to form PsyCap: self-efficacy, hope, optimism, and resilience. Based on research (e.g., Luthans and Youssef, 2004; Luthans et al., 2005), *PsyCap* can be defined as “a core psychological factor of positivity in general, and POB (Positive organizational behavior) criteria meeting states in particular, that go beyond human and social capital to gain a competitive advantage through investment/development of *who you are*” (Luthans et al., 2005, p. 253).

For a better understanding of PsyCap, it can be informative to look at the definitions of the sub-domains as well. In positive psychology, *hope* is often defined as the perceived capability to obtain pathways to desired goals and motivate oneself via agency thinking to use those pathways (Luthans et al., 2005). *Self-efficacy* can be understood as confidence in one’s own ability to achieve one’s goals and high levels of performance through motivational and cognitive resources as well as the pursued course of action. The concept of self-efficacy is based on the social-cognitive theory developed by Bandura (1977, 1992, 1997, 2001). The theory states that amongst others, cognitive, emotional, and motivational processes are controlled through personal convictions. These contain expected action results, outcome expectancies, and perceived self-efficacy. *Optimism* refers to an individual’s anticipation of positive results (Scheier et al., 2001). *Resilience* addresses “the ability of an individual to bounce back from adversity, uncertainty, risk or failure, and adapt to changing and stressful life demands” (Lorenz et al., 2016, p. 2).

### 1.1 PsyCap

To grasp the meaning of PsyCap as a construct, not only the theoretical definition is important but also the incorporation of its implications for human resource development and its effects across different life domains into the personal understanding of the construct (Dudasova et al., 2021). Avey et al. (2011) state in their meta-analysis that amongst others there is a positive relationship between desirable employee attitudes (e.g., job satisfaction, organizational commitment, psychological wellbeing), behavior (i.e., citizenship), and PsyCap. Furthermore, there are negative relationships between PsyCap and undesirable employee attitudes (e.g., cynicism, turnover intentions, job stress, and anxiety) and behavior (i.e., deviance). Like other domains, PsyCap is linked to mental health (e.g., Selvaraj and Bhat, 2018). There are multiple theoretical mechanisms postulated to explain the effect of PsyCap on wellbeing (Youssef-Morgan and Luthans, 2015).

These mechanisms include “psychological resource management cognitive and affective appraisals and memory retention processes” (Yang et al., 2021, p. 3). Evidence suggests that positive emotions are related to better physical and psychological health (Tugade et al., 2004). Positive affect is often associated with optimism and the likelihood of people to keep up positive prospects in times of adversity (Lyubomirsky et al., 2005). Together with other research supporting similar connections, an undeniable link between PsyCap and positive affect is evident in the literature (e.g., Lorenz et al., 2016). In this context, the assumption that PsyCap is beneficial for mental and physical health is not far-fetched. Individuals with higher PsyCap are able to better cope with stress (Avey et al., 2009). In other words, PsyCap serves as a protective buffer against stress and promotes one’s psychological and physical wellbeing (Baron et al., 2016).

### 1.2 Measuring psychological capital

To assess PsyCap, the 24-item Psychological Capital Questionnaire (PCQ-24) was developed by Luthans et al. (2007). Subsequently, the short version of the original PCQ–24, PCQ–12, consisting of 12 items was developed. Although Luthans et al. empirically validated both PCQ instruments and they are widely used since, several issues of concern are raised by critical evaluations (e.g., Dawkins et al., 2013; Newman et al., 2014). Primarily, thus far no consensus is reached on the internal structure of the PCQ–24 (e.g., Görgens-Ekermans and Herbert, 2013; Sahoo and Sia, 2015) nor for the PCQ-12 (e.g., Luthans et al., 2008; Caza et al., 2010; Avey et al., 2011). Other alternative measures such as the semi-projective Implicit Psychological Capital Questionnaire (I-PCQ) by Harms and Luthans (2012) also raise concerns related to the psychometric properties of the instrument. In addition to the concerns related to the test-retest reliability and convergent and discriminant validity (Dawkins et al., 2013), the cultural differences seem to affect the results reported in various international studies (Wernsing, 2014; Sahoo and Sia, 2015). In particular, the lack of availability of psychometric evaluations of some translated PCQ scales is a cause of concern. Therefore, Dudasova et al. (2021) expressed the need for cautious interpretations of findings derived from studies utilizing the PCQ scales given the existence of certain evidence of poor adaptations and psychometric deficiencies. Another criticism of the PCQ scales is the low availability of open access, limiting the application in small organizations and other non-business organizational contexts (Lorenz et al., 2016).

### 1.3 The Compound PsyCap Scale

To address the existing discrepancies in PCQ scales and to provide a validated open access licensed assessment scale, Lorenz et al. (2016) developed the Compound PsyCap Scale (CPC–12) consisting of 12 total items and an equally balanced distribution of items per subscale (four subscales with three items each). Although the original validation study (Lorenz et al., 2016) and the subsequent studies utilizing the CPC-12 presented evidence of strong psychometric properties (e.g., Khajavy et al., 2019). The CPC–12 was recently adapted due to a statistical overlap between the factors of resilience and self-efficacy in a re-analysis among different samples (Dudasova et al., 2021). The revised version (CPC–12R), validated with Czech samples, consisted of nine original statements and three new resilience items strengthening the difference between the subscales assessing self-efficacy and resilience.

### 1.4 Aim of study

The first aim of this study was to test the psychometric properties of the German version of the CPC-12R in a German sample, and to validate an English version of the CPC–12R with an English-speaking US sample. Furthermore, to enable the use of the revised CPC–12R in studies comparing PsyCap in English and German-speaking samples, we also tested the level of measurement invariance between the samples from both countries. To test the external validity of the CPC–12R, we selected several psychological constructs similar to those utilized in the testing of CPC–12 (Lorenz et al., 2016): satisfaction with life, and gratitude, as well as other constructs: wellbeing, prosocial behavior, perceived stress, and mental distress. Therefore, we expect the CPC-12R to be positively correlated with the positive mental health measures (i.e., satisfaction with life, gratitude, wellbeing, prosocial behavior) and negatively correlated with all negative measures (i.e., perceived stress and mental distress).

### 1.5 Satisfaction with life

Although studies on PsyCap often concentrate on work-related outcomes (Newman et al., 2014), work and non-work life influence each other (Ford et al., 2007). Furthermore, Ford et al. (2007) suggest time-based pressure as a major reason for such mutual interaction. Newman et al. (2014) also stated that in addition to predicting higher levels of work-family conflict, low PsyCap predicts less meaning of life which subsequently results in a lower life satisfaction. These results align with the reports that life satisfaction is positively related to optimism and self-esteem (Lucas et al., 1996). Thus, there is evidence that satisfaction with life and PsyCap are interrelated (Dirzyte et al., 2021). Therefore, a positive correlation between PsyCap and satisfaction with life can be expected.

### 1.6 Gratitude

Research has shown a close connection between PsyCap and gratitude (Luthans et al., 2006). Gratitude is seen as a promising indicator for inclusion and described as “the extra mile willingly traveled [*sic*] by those with high PsyCap” (p.180). Among other coherences, the positive correlations between gratitude and hope (*r* = 0.18–0.67) as well as between gratitude and optimism (*r* = 0.28–0.58) (McCullough et al., 2002), a positive correlation between gratitude and PsyCap seem reasonable.

### 1.7 Wellbeing

According to Seligman (2011), wellbeing constitutes of five pillars, namely Positive Emotions (P), Engagement (E), Positive Relationships (R), Meaning (M), and Accomplishment (A) (denoted by PERMA acronym). The PERMA-Profiler 15 (PP) questionnaire measures all five independent domains of wellbeing (Butler and Kern, 2016). In previous studies, PsyCap’s strong positive association with wellbeing is well established (Avey et al., 2009; Culbertson et al., 2010), in particular between PsyCap and each of the PERMA factors of wellbeing (Selvaraj and Bhat, 2018). Thus, a positive correlation between wellbeing and PsyCap is expected.

### 1.8 Prosocial behavioral intention

Prosocial behavior refers to “the ways in which people voluntarily and intentionally help other people” and prosocial intentions “reflect” “a person’s readiness to help others” (Baumsteiger and Siegel, 2019, p. 1). There is evidence that prosocial behavior is in different ways associated with subjective wellbeing, affect, personal resilience, and depression and anxiety (Zhao et al., 2020). As deduced above, all these constructs are connected to PsyCap, suggesting a positive relationship between prosocial behavior and or intention and PsyCap.

### 1.9 Mental distress

Several studies (e.g., Thomsen, 2006; Aldao et al., 2010), propose that a higher or dysregulated negative affect is associated with depressive symptoms and could predict the onset of depression (Nolen-Hoeksema et al., 2008; Bos et al., 2013). Thus, positive correlations between NA and depression as well as anxiety (Hofmann et al., 2012) can be expected. The relationship between positive affect and PsyCap becomes very clear when reviewing the literature which often relates positive affect to three major components also included in PsyCap: resiliency, self-efficacy, and optimism (Luthans et al., 2007).

Research has shown that people with high positive affect often resolve problems in a more effective way, show more mature coping efforts, and experience less conflicts at work (Lyubomirsky et al., 2005). Furthermore, positive correlations between all four components of PsyCap and positive affect (*r* = 0.28–0.68) is previously reported (Lorenz et al., 2016). positive affect and negative affect can be conceptualized as diametrically opposed. In agreement with that, Lyubomirsky et al. (2005) propose that negative affect and positive affect “regularly show moderate inverse relations across individuals, justifying the use of such negative states as the inverse of positive affect” (p. 822). This resonates with research that has found PsyCap having negative effects on states like stress and anxiety (Baron et al., 2016). All this leads to the conclusion that depression, anxiety, and negative affect can be expected to correlate negatively with PsyCap.

### 1.10 Perceived stress

Perceived stress refers to the degree to which events in a person’s life are assessed as stressful, unpredictable and uncontrollable (Cohen et al., 1983). Several studies in the literature show PsyCap negatively being associated with stress (e.g., Avey et al., 2009; Maykrantz et al., 2021). PsyCap is therefore regarded as a resource to buffer against heightened stress during uncertainties and psychological distress. Perceived stress can be expected to correlate negatively with PsyCap.

## 2 Materials and methods

The US sample consisted of 385 participants (*M*_*age*_ = 31.84 years, *SD*_*age*_ = 10.36) and the German sample consisted of 202 participants (*M_*age*_* = 35.99 years, *SD*_*age*_ = 14.99). All participants were over the age of 18 and employees at different workplaces. In the US sample, 52.5% of participants identified as male, 46.8% as female, and the remaining 0.5% as non-binary. In the German sample, 44.6% identified as male and 55.4% as female. In the US sample, 193 participants reported full-time employment and 94 part-time employment. In the German sample, 94 participants reported full-time employment and 73 part-time employment. The educational level was mostly bachelor education in the US sample (51.2%), followed by some master’s level education (24.7%). In the German sample, 27.2% reported having a university degree and 13.9% a technical or occupational certificate.

### 2.1 Participant recruitment and survey

For each population the surveys were conducted in the respective native language. The American survey was autonomously assessed by *Amazon Mechanical Turk*. For the German survey recruitment was mostly online based, on social media advertisement as well as advertisement in online magazines and on the website of the *Deutschsprachiger Dachverband für Positive Psychologie e.V*. The surveys consisted of demographic data, the CPC–12R scale (Dudasova et al., 2021) and the following instruments or their German translations, respectively: PSS-10, PP, Satisfaction With Life Scale (SWLS), Patient Health Questionnaire (PHQ–4), Prosocial Behavioral Intentions Scale (PBIS), and the Gratitude Questionnaire—Six Item Form (GQ–6). The Participants were informed that by proceeding past the welcome page of the online survey they would give their consent.

### 2.2 Instruments

Participants were asked to state their age, gender, highest level of completed education, employment status, whether they are currently enrolled at a university and if so, which degree they are aiming to achieve.

#### 2.2.1 Psychological capital

PsyCap was assessed using a revised version of the Compound PsyCap Scale—12 (CPC-12R; Lorenz et al., 2016; Dudasova et al., 2021). Three items each assessed the four subscales “hope,” “optimism,” “resilience,” and “self-efficacy” with a higher-order factor “PsyCap” using a 6-point rating scale ranging from 1 = “*strongly disagree*” to 6 = “*strongly agree.*” The complete list of items in German and English is shown in Table 1. A higher score indicates a higher level of PsyCap. In the current study, Cronbach’s alpha was 0.91 in the German sample and 0.90 in the US sample. McDonalds omega ω_*t*_ was 0.94 in the German sample and 0.92 in the US sample.

**TABLE 1:** Compound PsyCap Scale items of the German and English version.

Item label	Item
H1	Sollte ich mich in einer Zwickmühle befinden, würden mir viele Auswege einfallen. If I should find myself in a jam, I could think of many ways to get out of it.
H2	Im Moment betrachte ich mich als recht erfolgreich. Right now, I see myself as being pretty successful.
H3	Mir fallen viele Strategien ein, um meine derzeitigen Ziele zu erreichen I can think of many ways to reach my current goals.
E1	In unerwarteten Situationen weiß ich immer, wie ich mich verhalten soll I am confident that I could deal efficiently with unexpected events.
E2	Wenn ein Problem auftaucht, kann ich es aus eigener Kraft meistern. I can solve most problems if I invest the necessary effort.
E3	Schwierigkeiten sehe ich gelassen entgegen, weil ich mich immer auf meine Fähigkeiten verlassen kann. I can remain calm when facing difficulties because I can rely on my coping abilities.
R1	Ich glaube, dass ich sehr viel aushalte, ich lasse mich durch Misserfolge nicht leicht entmutigen. I consider myself to be able to stand a lot, I am not easily discouraged by failure.
R2	Ich neige dazu, mich von ernsthaften Lebensschwierigkeiten schnell wieder zu erholen. After serious life difficulties, I tend to quickly bounce back.
R3	Ich glaube, dass die Bewältigung von Stress mich stärken kann. I believe that coping with stress can strengthen me.
O1	Ich freue mich auf das Leben, das noch vor mir liegt. I am looking forward to the life ahead of me.
O2	Die Zukunft wird für mich viel Gutes mit sich bringen. The future holds a lot of good in store for me.
O3	Alles in allem erwarte ich, dass mir mehr gute als schlechte Dinge widerfahren. Overall, I expect more good things to happen to me than bad.

The abbreviations H1, H2, H3, E1, E2, E3, R1, R2, R3, O1, O2, and O3 represent the items of the CPC-12R. The Letters of the abbreviations indicate which is the respective latent factor, H, hope; E, self-efficacy; R, resilience; and O, optimism. The first line shows the German item and the second line shows the respective item in English.

#### 2.2.2 Satisfaction with life

Life satisfaction was assessed using the SWLS (Diener et al., 1985; Glaesmer et al., 2011). On a 7-point rating scale ranging from 1 = “*strongly agree*” to 7 = “*strongly disagree*,” participants rated five statements, such as “I am satisfied with my life.” A higher score indicates greater satisfaction with life. In the current study, Cronbach’s alpha was 0.88 in the US sample and 0.86 in the German sample. McDonalds omega ω_*t*_ was 0.87 in the German sample and 0.89 in the US sample.

#### 2.2.3 Gratitude

Gratitude was assessed using the Gratitude-Questionnaire (GQ-5; Hudecek et al., 2020). Given a 7-point rating scale ranging from 1 = “*strongly disagree*” to 7 = “*strongly agree*” participants were asked to rate how much they agreed with the presented statements (e.g., “I have so much in life to be thankful for”). A higher total score indicates higher gratitude. In the current study, Cronbach’s alpha was 0.75 in the US sample and 0.79 in the German sample. McDonalds omega ω_*t*_ was 0.81 in the German sample and 0.80 in the US sample.

#### 2.2.4 Wellbeing

Wellbeing was assessed using the PERMA-Profiler (Butler and Kern, 2016; Wammerl et al., 2019), which uses an 11-point rating scale ranging from 0 = *“never”* to 10 = *“always”* or 0 = *“not at all”* to 10 = *“completely.”* This scale is based on Seligman’s (2011) wellbeing theory that comprises five building blocks of wellbeing, namely positive emotions (i.e., experiencing happiness, joy, gratitude, etc.), engagement (i.e., using one’s strengths to meet challenges and experiencing flow), relationships (i.e., connecting with others and positive connections), meaning (i.e., finding one’s purpose, connecting to meaning), and accomplishment (i.e., pursuing and accomplishing goals). In addition to the 15 PERMA items (e.g., “How often do you become absorbed in what you are doing?”), the instrument includes eight filler items. A higher overall score indicates a higher level of wellbeing. In the current study, Cronbach’s alpha was 0.92 in the US sample and 0.94 in the German sample. McDonalds omega ω_*t*_ was 0.97 in the German sample and 0.97 in the US sample.

#### 2.2.5 Prosocial behavior

Prosocial behavior was assessed using the Prosocial Behavior Intention Scale (PBIS, Baumsteiger and Siegel, 2019), using a 7-point rating scale ranging from 1 = *“Definitely would not do this”* to 7 = *“Definitely would do this.”* Participants rated four given statements (e.g., “Comfort someone I know after they experience a hardship”). A higher score indicates a higher level of prosocial behavior. In the current study, Cronbach’s alpha was 0.82 in the US sample and 0.66 in the German sample. McDonalds omega ω_*t*_ was 0.69 in the German sample and 0.82 in the US sample.

#### 2.2.6 Perceived stress

We assessed stress with the Perceived Stress Scale (PSS-10; Cohen et al., 1983; Reis et al., 2019), using a 5-point rating scale ranging from 1 = *“never”* to 5 = *“very often.”* Participants rated 10 given statements (e.g., “In the last month, how often have you felt nervous and stressed?”). A higher score indicates a higher level of perceived stress. In the current study, Cronbach’s alpha was 0.71 in the US sample and 0.89 in the German sample. McDonalds omega ω_*t*_ was 0.91 in the German sample and 0.84 in the US sample.

#### 2.2.7 Mental distress

Mental distress (depression and anxiety) was assessed using the Patient Health Questionnaire-4 (PHQ-4; Kroenke et al., 2009; Tibubos and Kröger, 2020) using a 4-point rating scale ranging from 1 = *“not at all”* to 4 = *“nearly every day.”* Participants rated four given statements (e.g., “Feeling down, depressed, or hopeless”). A higher total score indicates a higher level of psychological distress. In the current study, Cronbach’s alpha was 0.88 in the US sample and 0.86 in the German sample. McDonalds omega ω_*t*_ was 0.89 in the German sample and 0.90 in the US sample.

### 2.3 Analysis

One of the declared objectives of this study is the factorial validity of the CPC-12R, respectively, the model underlying the instrument. Several confirmatory factor analyses (CFAs) with a maximum likelihood robust estimation (MLR) were conducted to test the model of the CPC-12R. The MLR was chosen to prevent biased results caused by violations of the normality distribution (e.g., Beauducel and Herzberg, 2006). The model represents 12 items, four correlated first-order factors (hope, self-efficacy, optimism, and resilience) and one second-order factor. To test the fit of the measurement model, the criteria proposed by Hu and Bentler (1999) were used. Beyond χ^2^ significance testing, these criteria comprise a standardized root-mean-square residual (SRMR) ≤ 0.08 in combination with at least one of the following fit indices: a root-mean-square error of approximation (RMSEA) ≤ 0.06, a lower bound of the 90% confidence interval of the RMSEA ≤ 0.06, a comparative fit index (CFI) ≥ 0.95, or a Tucker Lewis index (TLI) ≥ 0.95. For the conduction of the CFAs, the *lavaan* package (version 0.6–9; Rosseel, 2012) of *R* statistical software (version 3.6.1; R Core Team, 2014) was used. Other used packages were *readxl* (version 1.3.1; Wickham and Bryan, 2019), *semTools* (version 0.5–5; Jorgensen et al., 2021), *semPlot* (version 1.1.2; Epskamp, 2019), and *psych* (version 2.1.9; Revelle, 2021a).

Due to forced choice in the standardized questionnaires, there was no missing data in the main analysis. As part of the descriptive data, two estimates of internal consistency were reported: Omega total (ω_*t*_) (McDonald, 1999), as well as Cronbach’s coefficient alpha (α) (Cronbach, 1951). Zinbarg et al. (2005) as well as Revelle and Zinbarg (2009) conclude in their comparative studies that ω_*h*_ is the best estimate of the general factor solution of a test. The ω_*t*_ was reported as an estimate of the total reliability of a test especially for tests that do not meet the assumption of τ-equivalence (Revelle and Condon, 2019). Since ω_*h*_ only applies to models that have a g-factor, it was not reported for the SWLS, the PBIS and the GQ-6.

Furthermore, because of α being used very often as a measure of internal consistency, it can be of use being reported, if only for reasons of comparability. In any case, Revelle (2021b) suggests routinely reporting all three measures named above. The *alpha* function of the R-package *psych* was used to estimate α. For the estimation of ω_*h*_ and ω_*t*_ the function *omegaSem* of the R-package *lavaan* was used.

The other main objective of this study is the testing of MI across different cultural backgrounds. For MI testing, the two samples were tested in one model using a multigroup confirmatory factor analysis (MGCFA) for the CPC-12R. The procedure of the MGCFA was inspired by Rudnev et al. (2018) as well as by Chen et al. (2005). The MGCFA was conducted using MLR as the estimator. As a prerequisite for the MGCFA on second-order level, the levels of invariance (viz., configural, metric, scalar, and residual invariance) were tested for the first-order factors aforehand. To test the MI, the criteria proposed by Cheung and Rensvold (2002) and those from Chen (2007) were used. Cheung and Rensvold declare that a ΔCFI larger than −0.01 should lead to the null hypothesis of invariance being rejected. Chen proposes that because of the influence different model-parameters have on the fit indices, the cut-offs of those fit indices should be adjusted in line with the parameters. Additionally, some influences apply especially in certain levels of invariance testing. Thus, the cut-offs should be also adjusted to the levels of invariance testing.

In this study, for MI testing with an adequate sample size (total *N* > 300), unequal sample sizes and mixed lack of invariance, the following cut-offs proposed by Chen (2007) were applied: For testing loading invariance, a change of ≥ −0.01 in the CFI, in addition with a change of ≥ 0.015 in RMSEA, or a change of ≥ 0.03 in SRMR indicates non-invariance. When testing intercept or residual invariance, a change of ≥ −0.01 in CFI, supplemented by a change of ≥ 0.015 in RMSEA, or a change of ≥ 0.01 in SRMR indicates non-invariance.

## 3 Results

The model fit indices for the German sample as well as for the US sample are displayed in Table 2. Indices of the model in the German sample that should be emphasized were the CFI = 0.97; TLI = 0.96; RMSEA = 0.07; 90% CI RMSEA = (0.05, 0.08), SRMR = 0.05. Respective values for the American sample were CFI = 0.98; TLI = 0.97; RMSEA = 0.05; 90% CI RMSEA = (0.03, 0.06), SRMR = 0.04.

**TABLE 2:** Confirmatory factor analyses and measurement invariance results.

Model	*X*^2^ (df)	Sig.	CFI	TLI	RMSEA [CI]	SRMR
CFA German sample	81.69 (50)	0.003	0.97	0.96	0.07 [0.46, 0.83]	0.05
CFA US sample	79.18 (50)	<0.001	0.98	0.97	0.05 [0.03, 0.64]	0.04
Configural invariance	160.72 (100)	<0.001	0.98	0.97	0.05 [0.04, 0.06]	0.04
Metric invariance	184.70 (111)	<0.001	0.97	0.97	0.05 [0.04, 0.07]	0.05
Scalar invariance	199.47 (118)	<0.001	0.97	0.97	0.05 [0.04, 0.07]	0.06

*X*^2^ refers to the Chi-square difference value with respective degrees of freedom (df).

Sig. is used to display the *p*-value of the Chi-square difference test. The confirmatory fit index is reported as CFI, the Tucker-Lewis index as TLI. RMSEA is the robust Root Mean Square Error of Approximation with respective 90%-confidence intervals [CI].

The SRMR is the Root Mean Square Residual. CFA means confirmatory factor analysis.

MGCFA means multigroup confirmatory factor analysis.

**FIGURE 1:**
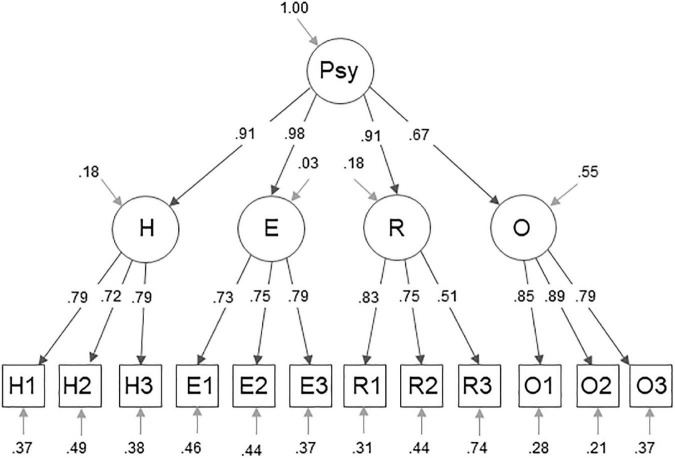
Four (plus g-) factor measurement model for PsyCap. This figure displays the measurement model of the CPC-12R within the German sample, showing standardized factor loadings and variances. The abbreviations H1, H2, H3, E1, E2, E3, R1, R2, R3, O1, O2, and O3 represent the items of the CPC-12R. The Letters of the abbreviations indicate which is the respective latent factor, hope (H), self-efficacy (E), resilience (R), and optimism (O). The abbreviation Psy refers to PsyCap.

**FIGURE 2:**
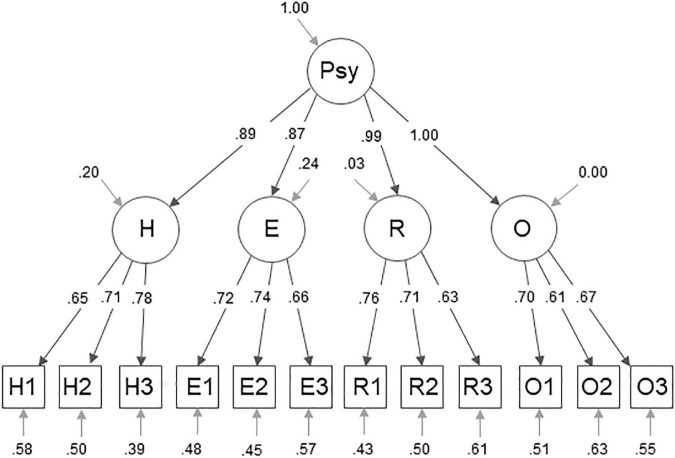
Four (plus g-) factor measurement model for PsyCap. This figure displays the measurement model of the CPC-12R within the US sample, showing standardized factor loadings and variances. The abbreviations H1, H2, H3, E1, E2, E3, R1, R2, R3, O1, O2, and O3 represent the items of the CPC-12R. The letters of the abbreviations indicate which is the respective latent factor, hope (H), self-efficacy (E), resilience (R), and optimism (O). The abbreviation Psy refers to PsyCap.

The measurement model of the German sample is displayed in Figure 1, the respective model for the American sample in Figure 2. The factor loadings in the German sample, that are to be highlighted, are the loadings of the second order factors on PsyCap: *H* = 0.91, *E* = 0.98, *R* = 0.91, and *O* = 0.67. Of the item loadings onto the first-order factors only the loading of the item R3 = 0.51 on the Resilience factor is to be emphasized. The loadings to highlight in the US sample are *R* = 0.99 and *O* = 1.0. Furthermore, the item loadings onto the respective first-order factors of the items H1 = 0.65, E3 = 0.66, R3 = 0.63, O2 = 0.61, and O3 = 0.67 stand out.

Table 3 presents descriptive statistics, bivariate correlations with confidence intervals, Cronbach’s α and McDonald’s ω for the study variables in the US sample. Table 4 presents the same in the German sample. All correlations are according to our hypotheses (German values in brackets). PERMA [*r* = 0.78 (0.73)] and satisfaction with life [*r* = 66 (0.67)] showed the highest positive correlations with the CPC-12R. Perceived stress [*r* = −0.40 (−0.67)] and mental distress [*r* = −0.22 (−0.63)] showed statistically significant negative correlations with the CPC-12R in both samples.

**TABLE 3:** Means, standard deviations, estimates of internal consistency, and correlations with confidence intervals for the US sample.

Variable	α	ω_t_	*M*	*SD*	1	2	3	4	5	6
1. Life satisfaction	0.88	0.89	5.03	1.24						
2. PERMA	0.92	0.97	6.95	1.31	0.74***					
					[0.69, 0.78]					
3. Stress	0.71	0.84	2.92	0.59	−0.33***	−0.55***				
					[−0.42, −0.24]	[−0.61, −0.47]				
4. Pro-social intention	0.82	0.82	5.58	1.11	0.28***	0.47***	−0.13**			
					[0.19, 0.37]	[0.39, 0.54]	[−0.23, −0.03]			
5. Mental distress	0.88	0.90	2.32	0.87	−0.13*	−0.38***	0.67***	0.05		
					[−0.22, −0.03]	[−0.47, −0.29]	[0.61, 0.72]	[−0.05, 0.15]		
6. Gratitude	0.75	0.80	5.03	1.04	0.36***	0.55***	−0.43***	0.55***	−0.34***	
					[0.27, 0.44]	[0.48, 0.62]	[−0.51, −0.35]	[0.48, 0.62]	[−0.43, −0.25]	
7. PsyCap	0.90	0.92	4.61	0.79	0.66***	0.76***	−0.40***	0.46***	−0.22***	0.54***
					[0.60, 0.71]	[0.72, 0.80]	[−0.48, −0.31]	[0.38, 0.54]	[−0.31, −0.12]	[0.46, 0.61]

*M* and *SD* are used to represent mean and standard deviation, respectively. Values in square brackets indicate the 95% confidence interval for each correlation. The confidence interval is a plausible range of population correlations that could have caused the sample correlation (Cumming, 2014).

*Indicates *p* < 0.05, **indicates *p* < 0.01, ***indicates *p* < 0.001. α indicates Cronbach’s coefficient alpha (Cronbach, 1951) and ω_t_ indicates omega total (McDonald, 1999).

**TABLE 4:** Means, standard deviations, estimates of internal consistency, and correlations with confidence intervals for the German sample.

Variable	α	ω_t_	*M*	*SD*	1	2	3	4	5	6
1. Life satisfaction	0.86	0.87	4.73	1.27						
2. PERMA	0.94	0.97	7.02	1.44	0.82***					
					[0.77, 0.85]					
3. Stress	0.89	0.91	2.71	0.72	−0.63***	−0.71***				
					[−0.70, −0.55]	[−0.76, −0.64]				
4. Pro-social intention	0.66	0.69	6.00	0.83	0.11	0.23***	−0.12			
					[−0.01, 0.24]	[0.10, 0.34]	[−0.25, 0.00]			
5. Mental distress	0.86	0.89	1.98	0.76	−0.63***	−0.74***	0.79***	−0.10		
					[−0.70, −0.54]	[−0.80, −0.68]	[0.74, 0.83]	[−0.23, 0.03]		
6. Gratitude	0.79	0.81	5.58	0.98	0.60***	0.67***	−0.38***	0.25***	−0.41***	
					[0.52, 0.68]	[0.59, 0.73]	[−0.49, −0.27]	[0.13, 0.37]	[−0.51, −0.29]	
7. PsyCap	0.91	0.94	4.32	0.81	0.68***	0.78***	−0.67***	0.18**	−0.63***	0.54***
					[0.61, 0.75]	[0.72, 0.82]	[−0.73, −0.59]	[0.05, 0.30]	[−0.70, −0.54]	[0.44, 0.62]

*M* and *SD* are used to represent mean and standard deviation, respectively. Values in square brackets indicate the 95% confidence interval for each correlation. The confidence interval is a plausible range of population correlations that could have caused the sample correlation (Cumming, 2014).

*Indicates *p* < 0.05, **indicates *p* < 0.01, ***indicates *p* < 0.001. α indicates Cronbach’s coefficient alpha (Cronbach, 1951) and ω_t_ indicates omega total (McDonald, 1999).

Parameters that are to be highlighted are the α = 0.66 of the PBIS in the German sample, as well as the α = 0.82 in the American sample. The PP showed α = 0.94 in the German sample and α = 0.92 in the US sample. Also, to be mentioned are the α = 0.91 of the CPC-12R in the German sample and the American α = 0.90. Respectively, the ωt = 0.94 of the German and the ωt = 0.92 of the American sample should be highlighted as well. Values that should be considered additionally are ω_*h*_ = 0.06 of the PSS in the American sample and ω_*t*_ = 0.69 of the PBIS in the German sample.

The resulting fit indices of the test for configural invariance, metric invariance and scalar invariance are displayed in Table 2. Indices of the second-order MGCFA, that should be emphasized, are the CFI = 0.97; TLI = 0.97; RMSEA = 0.05; 90% CI RMSEA = (0.04, 0.06) and SRMR = 0.05, regarding the metric invariance. Furthermore, the CFI = 0.97; TLI = 0.97; RMSEA = 0.05; 90% CI RMSEA = (0.04, 0.07) and SRMR = 0.06.

## 4 Discussion

The results of the CFA for both the German and the US sample indicate that, according to the criteria proposed by Hu and Bentler (1999), the model of the CPC-12R fits the data well. Although the chi-square significance tests were significant in both cases, this can be interpreted as a good fit because of the bias toward significance for large sample sizes. In the previous study of Dudasova et al. (2021), there were problems regarding the content selectivity or the redundancy of the two factors resilience and self- efficacy of the examined PsyCap scale. This could be seen for example in the similar, high factor loadings. Aiming to address this issue, the residual-covariance matrix of the German sample was examined but did not show similar values in the concerned items. Thus, redundancy is not indicated (see Table 5). The US sample, however, had similar values of the items R1, R2, and E1 (see Table 6). This could suggest substantive similarity of those items. The second-order factor loadings in the CFAs in both samples were high overall, indicating good prediction of PsyCap by the proposed factors. While several second-order factor loadings showed values close to 1.0, the first-order loadings overall were more moderate but still indicate good measurement of the factors through the chosen items. Exceptions are the item R3 in both samples and the items H1, E3, R3, O2, and O3 in the US sample. Those items showed comparatively low factor loadings, indicating that they might not be the best items to measure or predict the respective latent factors.

**TABLE 5:** Residual-covariance matrix of the CPC-12R in the German sample.

Items	H1	H2	H3	E1	E2	E3	R1	R2	R3	O1	O2
2. H2	-0.04										
3. H3	-0.02	0.08									
4. E1	0.11	-0.06	0.06								
5. E2	0.06	-0.01	-0.04	-0.02							
6. E3	0.02	-0.07	-0.03	0.03	-0.01						
7. R1	0.02	-0.04	-0.06	0.01	0.03	0.05					
8. R2	-0.06	-0.01	-0.05	-0.05	-0.04	0.01	-0.01				
9. R3	-0.01	-0.01	-0.08	0.02	0.01	-0.03	-0.05	0.08			
10. O1	0.01	0.14	0.07	-0.12	0.07	0.03	0.10	0.20	0.13		
11. O2	-0.07	0.03	0.04	-0.16	-0.02	-0.07	-0.06	0.07	0.04	0.01	
12. O3	-0.02	0.12	0.01	-0.11	0.05	-0.03	0.04	0.14	0.09	-0.04	0.02

The abbreviations H1, H2, H3, E1, E2, E3, R1, R2, R3, O1, O2, and O3 represent the items of the CPC-12R.

The letters of the abbreviations indicate which is the respective latent factor, H, hope; E, self-efficacy; R, resilience; and O, optimism.

**TABLE 6:** Residual-covariance matrix of the CPC-12R in the US sample.

Items	H1	H2	H3	E1	E2	E3	R1	R2	R3	O1	O2
2. H2	-0.04										
3. H3	-0.03	0.05									
4. E1	0.01	-0.02	0.06								
5. E2	0.04	-0.05	-0.01	0.05							
6. E3	-0.02	0.06	0.04	-0.06	-0.02						
7. R1	0.02	0.01	-0.03	-0.02	0.01	0.09					
8. R2	-0.01	-0.03	0.01	-0.02	-0.07	0.01	0.01				
9. R3	-0.01	-0.07	-0.06	0.03	0.09	0.04	-0.02	0.02			
10. O1	0.05	0.02	-0.04	-0.02	0.02	-0.09	0.01	0.01	0.04		
11. O2	0.11	-0.03	0.02	0.08	-0.01	0.06	-0.06	-0.08	-0.03	0.02	
12. O3	0.05	-0.01	-0.02	-0.11	-0.15	0.08	0.03	0.10	-0.01	-0.03	0.01

The abbreviations H1, H2, H3, E1, E2, E3, R1, R2, R3, O1, O2, and O3 represent the items of the CPC-12R.

The letters of the abbreviations indicate which is the respective latent factor, H, hope; E, self-efficacy; R, resilience; and O, optimism.

### 4.1 External validity

In the German sample the correlations of PsyCap with perceived stress, life satisfaction, gratitude, mental distress, wellbeing, and prosocial behavior were as expected. The correlations, displayed in Table 4, are in line with previous research on PsyCap which indicates good construct validity of the CPC-12R. Only the weak correlation of prosocial behavior with the CPC-12R was somewhat unexpected. As the Pro-Social Behavior Intention Scale previously has mainly been used in English, this could be due to the fact that the items of the scale do not work the same in a German-speaking sample as they do in an English-speaking sample. In the US sample the correlations of PsyCap with perceived stress, life satisfaction, gratitude, mental distress, wellbeing, and prosocial behavior turned out as it was anticipated. Those results (see Table 3) speak for the construct validity of the CPC-12R in the US sample.

### 4.2 Internal validity

Because α was mainly reported to facilitate comparison with existing studies, the individual values will not be investigated that excessively. However, the rather low α of the PBIS (α = 0.66) found in the German sample needs to be addressed. This α does not indicate appropriate internal validity and therefore could be a sign of bad internal consistency of the PBIS. Thus, it makes it questionable whether this scale should be taken for measuring the discriminant validity of the CPC-12R and prosocial behavioral intention. In the US sample this difference in the α scores was not found. The respective α in the US sample (α = 0.82) was in line with the α score of Baumsteiger and Siegel (2019). Furthermore, it must be noted that the α scores of the CPC-12R as well as those from the PP (see Tables 3, 4) are considerably high. Regarding the PP, this might in parts be due to the length of the scale. However, in both cases this should be seen as a sign of redundancy and content repetition across the included items because the scores are higher than α = 0.90 (Streiner, 2003). Schermelleh-Engel and Gäde (2020) suggest that omega coefficients should have a minimum value of 0.70 or even better should be in the 0.80–0.90 range. In addition, the CI should be narrow.

However, those guidelines apply to unidimensional models and equivalents for multidimensional models are yet to be specified. According to the specified cut-offs, the omega values indicate good model-based reliability. The exceptions are the ω_h_ = 0.06 of the PSS in the US sample and the ω_t_ = 0.69 of the PBIS in the German sample. Those results are signs that in the US sample the g-factor of the PSS is not the explanation of the true variance or that the portion that is explained is rather small. Furthermore, it is indicated that the portion of the total true variance that is contained in the total variance of the PBIS is not as large as it would be desirable. Because the cut-offs for values such as alpha and omega are arbitrary in most cases, comparative scores of previous studies were also used for the evaluation of the omega scores. Dudasova et al. (2021) found the overall ω_t_ of PsyCap measured by the CPC-12R to be ω_t_ = 0.89. Both the German (ω_t_ = 0.94) and the US (ω_t_ = 0.92) sample scored slightly higher in this study. Thus, they appear in line with the previous research indicating good internal validity.

### 4.3 Measurement invariance

The results of the MGCFA indicate that there is configural, metric as well as scalar invariance between the two samples. This can be concluded because the fit indices are changing within the range that was proposed by Chen (2007). But because it is also emphasized that fit indices are more a rough guideline than precise and generalizable cut-offs, the parameters were also investigated from different angles. It was considered whether the fit indices indicate a good model-fit according to Hu and Bentler (1999), as was the difference between the (robust) confirmatory fit indices being smaller than 0.01 (Cheung and Rensvold, 2002). When configural invariance exists, it means participants of different groups understand the constructs in the same way (Riordan and Vandenberg, 1994; Cheung and Rensvold, 2002). For the present study this means that the data assessed with the CPC-12R in the German sample as well as in the US sample consists of the same number of factors, with the same items included in each factor (Meredith, 1993; Cheung and Rensvold, 2002). In addition, metric invariance is also evident in the data, which indicates that all factor loading parameters are equal across groups (Cheung and Rensvold, 2002).

Specifically, this suggests that the items of the CPC-12R and their underlying constructs relate to the same strengths in both samples. Furthermore, scalar invariance could be established, suggesting that in addition to the factorial structure and the factor loadings, the vectors of the item intercepts are also invariant. Scalar invariance is necessary for the comparison of latent mean differences across groups, because it indicates that the scales that were used have the same unit of measurement as well as the same origin (Cheung and Rensvold, 2002; Chen et al., 2005). Residual or strict invariance assumes, additionally to the constraints above, that variable-residuals are equal across the groups. Here the residual invariance did not hold for the first-order factors and therefore could neither hold for the second-order model (Rudnev et al., 2018). As the residual variance did not hold for the CPC-12R, the items of the scale are not measuring the latent constructs with the same measurement error. This implies that it cannot be known whether differences measured by the items of the CPC-12R between the groups are due to group differences in the proposed factors or due to measurement errors (Cheung and Rensvold, 2002; Chen et al., 2005).

There are many possible reasons why the residual variance did not hold. Malpass (1977) suggests that amongst others, differences in vocabulary, grammar but also the usual experiences of different cultures can result in residual non-invariance. Although it is quite unlikely in this case, it should also be noted that if one of the compared groups is unfamiliar with a scale and the included scoring formats, this can lead to inconsistent response patterns and therefore to non-invariance (Mullen, 1995).

### 4.4 Outlook

Because it would go beyond the scope of this study, the MI was not explored further after the residual non-invariance had been met. However, this could be part of future research. As it is still currently under debate how measurement non-invariance should be treated when met, there are several options on how to proceed. For example, it was suggested by Byrne et al. (1989) that when a level of invariance is met, the respective constrained parameters exhibiting non-invariance when constrained should be set free. Meanwhile, those parameters that met the measurement invariance when constrained should be left constrained. Thus, partial invariance would be established.

Pohl and Schulze (2020) propose another approach to treat measurement non-invariance. Although they also suggest proceeding with partial invariance when met with non-invariance, the partial MI is achieved by defining a subset of items that meet the assumption of the respective level of MI. Those items are called anchor items and it is proposed to identify them using the *cluster approach* (Pohl and Schulze, 2020).

One distinct advantage of this approach is the possibility to choose from several item clusters the one that fits the best, or to report them all, in contrast to just getting one cluster of items that is not inevitably systematically assembled. In the absence of a starting point for future research, it could be beneficial to look at local model fit by means of modification indices (e.g., Thoemmes et al., 2018). Finally, it should be noted that with establishing partial MI, by adjusting the measurement model, one is giving up the confirmatory approach of using the CFA in favor of an exploratory, data-based approach.

### 4.5 Limitations

The results of this study should be interpreted with the following limitations in mind. First, the participants were recruited and participated online. Therefore, the study may not have reached a representative sample of individuals and thus lack generalizability. However, according to Gosling et al. (2004), the online recruitment should not yield major effects on the results. It should also be noted that the samples were not the same size and showed mixed lack of invariance. As explained by Chen (2007), this influences the MI testing and should be kept in mind. Even if adjusted cut-offs were being used, those only consider the general trends of influence that certain parameters have. Additionally, according to previous studies (e.g., Kass and Tinsley, 1979; Hu and Bentler, 1999) the sample sizes, although appropriate, could ideally have been larger. To address that issue, it could be a possible solution, to check the model fit using diagonally weighted least squares as an estimator instead of the MLR (Li, 2016). The German sample was rather small for conducting a CFA and could therefore have resulted in a smaller power of the procedure.

Furthermore, the samples were retrieved from German-speaking participants on the one hand and English-speaking participants on the other hand, thus the results mostly, if not exclusively, bear meaning for similar groups, samples, or applications. There also was a problem with specifying the number of latent factors in the *omegaSem* function of the PP in the German sample, so the respective omega values are to be regarded with caution. Finally, as mentioned in the discussion, the CPC-12R model did not exhibit residual invariance and is therefore limited in the extent conclusions should be drawn for practical application, based on the questionnaire.

## 5 Conclusion

In this study, we provide further validation for the revised version of the freely available CPC-12R. Future research validating this instrument in different languages and cultural contexts is encouraged. The present study is merely a first step in validating the CPC-12R in different countries including its factorial structure and measurement invariance. Additional research might incorporate data from other ethnically and culturally diverse regions like Africa, India, and South America. Furthermore, the standardization of the CPC-12R needs to be addressed. Contemporary and country-specific norms would be helpful for a meaningful interpretation of the actual scores of the instruments. This could help individuals gauge the extent to which they possess overall PsyCap, in addition to identifying aspects needing targeted PsyCap intervention. Overall, the obtained results indicate that the CPC-12R may constitute a useful tool for assessing the psychological capital in the German and US populations.

## Data availability statement

The datasets presented in this study can be found in online repositories. The names of the repository/repositories and accession number(s) can be found below: https://www.psycharchives.org/en/item/17049fb7-99c7-4ad9-bc0d-1f8302ec1197.

## Ethics statement

Ethical review and approval was not required for the study on human participants in accordance with the local legislation and institutional requirements. The patients/participants provided their written informed consent to participate in this study.

## Author contributions

TL and LH organized the database and performed the statistical analysis. All authors contributed to manuscript revision, read, and approved the submitted version, and contributed to conception and design of the study and wrote the first draft of the manuscript.
